# Plasma Volume Oscillations During Intravenous Infusion of Hyper-Oncotic Albumin

**DOI:** 10.3390/life15050749

**Published:** 2025-05-07

**Authors:** Robert G. Hahn

**Affiliations:** Department of Clinical Sciences at Danderyd Hospital, Karolinska Institutet, 182 88 Stockholm, Sweden; robert.hahn@ki.se; Tel.: +46-790745354

**Keywords:** blood, hemodilution, fluid, ringer, colloid, oscillations, Fourier analysis

## Abstract

Low-frequency oscillations of blood components have been observed when the plasma is diluted by crystalloid fluid. The present study explores whether oscillations also occur during the infusion of hyper-oncotic albumin 20%. For this purpose, the hemoglobin-derived plasma dilution, plasma colloid osmotic pressure, and plasma albumin concentration were measured on 15 occasions over 5 h in 72 volunteers. All of them received 3 mL/kg of albumin 20% over 30 min in various clinical settings. Quality checks excluded 35% of the concentration–time curves, leaving 137 for analysis. Fourier transforms applied to the residuals after curve-fitting showed that the dominating frequency was 144 ± 42 min (mean ± SD), corresponding to 0.007 Hz and a wave amplitude of 1.8 ± 0.9%. The highest percentile of the amplitudes corresponded to a “peak-to-peak” variation in the plasma volume by 6%, which corresponds to a fluctuation of 180 mL, or 45% of the maximum volume expansion following the infusion of albumin 20%. Differences between settings (volunteers, surgery, postoperative, and post-burn) were small. In conclusion, oscillations with very low frequency occurred after infusion of albumin 20%. They varied the plasma volume by 3.6% and by up to 6% in the percentile with the highest amplitudes. The oscillations are large enough to affect measurements of cardiovascular function.

## 1. Introduction

The vascular system is the subject of several oscillating rhythms [1]. Recently, plasma dilution occurring during intravenous fluid therapy was demonstrated to be associated with oscillations of the plasma volume with an average frequency of only 0.0003 hertz (Hz), which translates to a wave period length of 1 hour (h) [2]. Colloid fluids had longer dominating frequencies than crystalloid fluids. The amplitudes varied between 1% and 4% of the plasma volume and the “peak-to-peak” amplitude was up to 8%, which is large enough to confound physiological measurements.

The highest amplitudes were found when isotonic crystalloid fluid was administered during laparoscopic surgery, and the very highest were found when patients were placed in the Trendelenburg position. An analysis of fluid distribution during laparoscopy suggested that the lymphatic flow accelerates due to gravitational forces when the body is tilted in this way [2]. Current evidence suggests that the rapid flow of filtered fluid through the interstitial space and lymphatic system creates these plasma volume oscillations, but this is by no means clear.

Intravenous fluid therapy is a very common intervention in hospital. Crystalloid fluid is recommended as a routine treatment [3,4], but colloid solutions, of which albumin is the fluid of choice for many clinicians, offer selected benefits and may replace or supplement the use of crystalloids [5,6,7,8]. Importantly, the use of the hyper-oncotic albumin 20% reduces the risk of volume overload during surgery and intensive care because it expands the plasma volume more effectively than other fluids, which allows the infused volume to be on the low side [9,10].

The purpose of the present study was to explore plasma volume oscillations when albumin 20% is administered, which is a hyper-oncotic fluid. Three blood variables were measured, from which the residuals were analyzed after curve-fitting to a volume kinetic model. These variables were Hb-derived plasma dilution, the colloid osmotic pressure (COP), and the plasma albumin concentration.

The turnover of albumin 20% differs from crystalloid fluid in that the infused volume is quite small while its hyper-oncotic nature recruits extravascular fluid to the plasma. The hypothesis was that albumin 20% would induce smaller oscillations than crystalloid fluid, as the infused fluid volume is only 10% of what was previously infused of crystalloid fluid.

## 2. Materials and Methods

This report is a secondary publication to four studies in which 3 mL/kg of 20% albumin (approximately 200 mL) was administered over 30 min at a constant rate by an infusion pump to 72 subjects in the following clinical settings: healthy volunteers (N = 27) [11,12], post-burn patients (N = 15) [13], ongoing surgery with minor blood loss (N = 15) [14], and postoperative patients (N = 15) [15]. In addition, the same amount of albumin was administered as a 5% solution to 12 volunteers [11]. All studies were performed in a consistent way under the supervision of the author. The same protocol was used, and purpose was always to study the hemodilution during fluid therapy for subsequent kinetic analysis. Reporting adhered to the STROBE checklist.

### 2.1. Subjects

The healthy volunteers fasted from midnight but ingested one sandwich and drank one glass (200 mL) of clear liquid 2 h before blood sampling started at 7.00–9.00 AM. None of them used daily medication.

The post-burn study included in-hospital patients with a burn area measuring > 6% of their total body surface area and were studied for 7 days (range: 4–14) after the burn. They were deemed to be normovolemic when the study started.

The study of ongoing elective surgery with minor blood loss and no epidural anesthesia recruited patients scheduled for surgery lasting for at least 5 h but without an expected major hemorrhage. They fasted from midnight and were premedicated with 1 g of oral paracetamol. Surgeries included correction of pro- or retrognathia and tissue reconstruction after the removal of a breast due to cancer. Chronic medications taken included tamoxifen (N = 3) and goserelin (N = 1) to treat breast cancer, and sumatriptan (N = 1) for migraine. A low-dose infusion of norepinephrine (mean rate: 2.6 µg/min) was given intermittently at the clinician’s discretion to half the patients to maintain their arterial pressure.

Postoperative patients were studied in the early morning after having undergone lengthy open abdominal cancer surgery during the preceding day. Hemorrhages averaged 700 mL and were fully compensated during the operations, which lasted 5.9 h. The postoperative patients were subject to invasive hemodynamic monitoring and deemed to be normovolemic when the study started.

The post-burn and the postoperative patients had a moderately severe inflammatory reaction with plasma C-reactive concentrations between 60 and 90 mg/L. The other groups had normal C-reactive concentrations (<5 mg/L).

The exclusion criteria were age < 18 years, pregnancy, severe allergy, kidney failure, heart failure, blood coagulation problems, blood hemoglobin (Hb) concentration < 90 g/L, expected blood loss > 500 mL, and American Society of Anesthesiologist’s (ASA) physical status classes III-IV. Hence, no patient had severe cardiovascular disease or decreased kidney function. Detailed descriptions of the studied populations are given in the original publications [3,4,5,6].

### 2.2. Data Collection

A venous cannula was placed on the cubital vein on both arms, one for infusing fluid and the other for blood. Venous blood (10 mL) was withdrawn at 15 exactly timed occasions over a period of 5 h after starting the infusion (0, 10, 20, 30, 40, 50, 60, 75, 90, 120, 150, 180, 210, 240, and 300 min). This observation time was chosen because we assumed that it would agree with the half-life of the plasma volume expansion.

Whole blood was analyzed for Hb concentration and hematocrit (Hct). Plasma was used to measure the plasma albumin concentration. The respective hospital’s certified laboratory was used for all clinical chemistry analyses.

An Osmomat 050 (Gonotec, Berlin, Germany) was used to measure the plasma colloid osmotic pressure (COP) in our own research laboratory.

Coefficients of variation were approximately 1% (Hb) and 2% (albumin and COP). Data obtained after awaking from anesthesia were excluded.

The non-invasive mean arterial pressure (MAP) was measured in the arm not used for fluid infusion using an automatic device (Datex-Ohmeda, GE Healthcare, Las Vegas, NV, USA).

### 2.3. Curve-Fitting and Fourier Analysis

The data were used to create curves showing the fractional change in the Hb-derived plasma dilution, COP, and albumin concentration over time. The plasma dilution was calculated as [(Hb_baseline_/Hb_later_) − 1)]/(1 − hematocrit_baseline_). Each dilution underwent a minimal mathematical correction to account for surgical hemorrhage, if any, and blood sampling.

The plasma curves were created by deleting the Hct (hematocrit) term and reversing the nominator and denominator in the Hb term.

Three different kinetic models (absorption, one-compartment, and two-compartment) were fitted to all curves.

The goal was to take the best possible mathematical representation of the curve for analysis of the residuals using signal processing with the Fast Fourier Transform function (FFT) implemented in MATLAB R2023a (Math Works, Inc., Natick, MA, USA) [2]. Fourier transformation is a mathematical approach that converts time-domain signals into spectral components, thereby providing frequency information about the signal. The technique is widely used in audio processing and image filtering. In the present study, the data were interpolated to regular intervals and sampled at 0.0033 Hz due to the shortest sampling interval of 10 min. A quadratic equation was fitted to one or two of the most apparent residuals to determine their wave period and amplitude.

The fraction of the total frequency interval that could be regarded as an apparent residual was restricted to 30% due to the long interval between the measurements (“low frequencies”). This exclusion was implemented to prevent the consideration of residuals with very high frequencies, although the sum of both low and high frequencies could account as residuals. Similarly, the amplitude threshold was set to 0.7 times the oscillation with the highest amplitude, to exclude frequencies that caused minor variations in plasma dilution.

The output included the dominating frequency, its amplitude, and the fraction of the residuals that the high and low frequency bands accounted for. The finally reported results included oscillations that occupied >50% of the total residual area (i.e., including low frequencies). For completeness, the percentage of the residual area covered by the analysis is reported both with (>30%) and without (>50%) the low-frequency oscillations being included.

Data on the central body fluid volume (*V*_c_) expanded due to the infusion of albumin 20% was taken from the original studies [11,12,13,14].

### 2.4. Statistics

Demographics data are reported as the mean ± standard deviation (SD). Relationships between parameters were studied using simple linear regression where r = correlation coefficient. The three variables used for the calculation of oscillations (plasma dilution, COP, and albumin) were compared using a repeated-measures analysis of variance (ANOVA). Differences between subgroups were analyzed using a one-way ANOVA. Bonferroni’s correction was used to adjust for multiple testing. *p* < 0.05 was considered statistically significant.

## 3. Results

### 3.1. Plots of Oscillations

**Figure 1:**
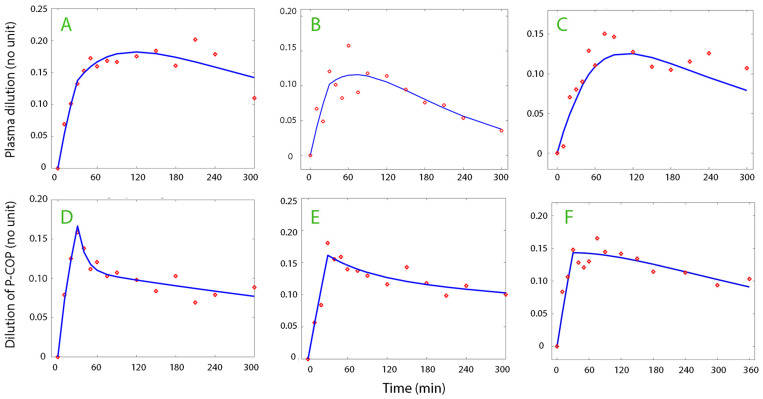
Examples of curve fits. Top row (**A**–**C**): Absorption kinetics (blue lines) fitted to plasma dilution (red circles). Bottom row: Two-volume (subplots **D** and **E**) and absorption kinetics (subplot **F**) fitted to the fractional increase in plasma colloid osmotic pressure (COP).

The most suitable kinetic model (absorption, one-compartment, or two-compartment) was used for the analysis of the plasma dilution–time, COP–time, and albumin–time curves. The absorption model was most often suitable for the plasma volume (Figure 1, top row) while the one- and two-compartment models were applicable for the plasma oncotic pressure and albumin data series (Figure 1, lower row). The data used for the kinetic analysis are shown in Supplementary Material.xls.

The curve-fitting was performed on 210 curves (1466 data points) but 46 curves were withdrawn from further processing because the subsequent Fourier analysis could not identify a dominant oscillation pattern. Twenty-seven additional curves were discarded as the fraction of the dominant frequency (without low-frequence bands) was <50% of the total residual area. Hence, 137 curves were analyzed.

**Figure 2:**
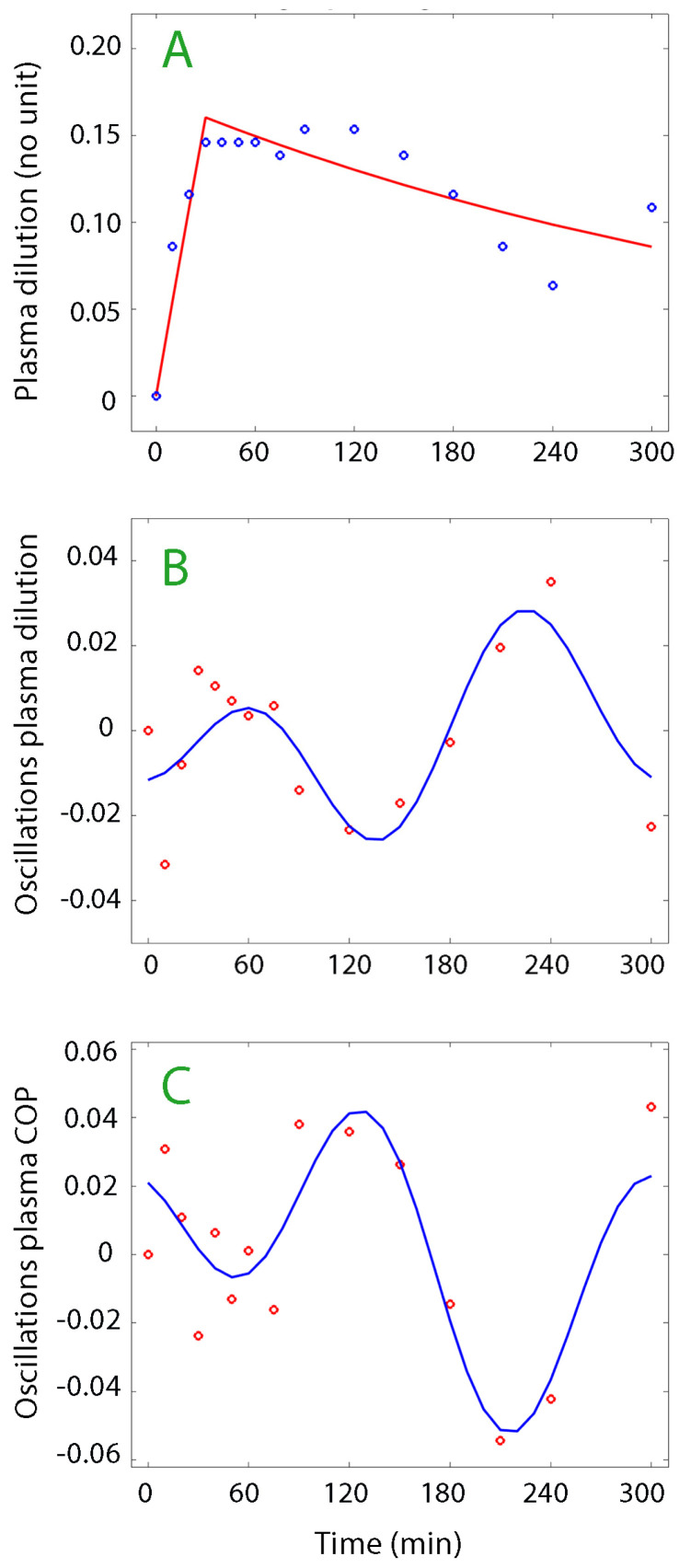
(**A**) Plasma dilution curve analyzed according to a one-volume kinetic model. Each point represents one measurement of plasma dilution and the red line the optimal curve fit. (**B**) Oscillations in the plasma dilution curve (Fourier analysis, blue line). (**C**) Oscillations in the curve for colloid osmotic pressure. Positive oscillations represent vascular contraction, but, for COP, they can also imply the number of molecules with colloid strength that have been added to the plasma. In subplots **B** and **C**, each red circle represents the residual of one measurement of plasma dilution after fitting the plasma dilution curve to the best of the three kinetic models, in this case being the one-compartment model.

**Figure 3:**
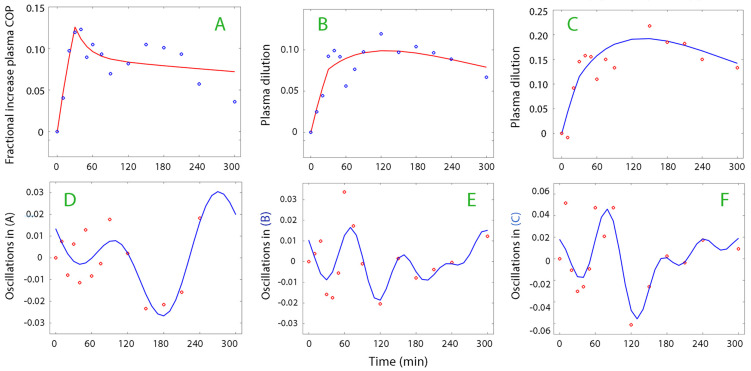
(**A**) Fractional increase in plasma COP during surgery, analyzed according to a two-compartment model. (**B**) Plasma dilution in a post-burn patient, absorption model. (**C**) Plasma dilution in another post-burn patient, absorption model. (**D**) Fourier analysis of the residuals in plot (**A**). (**E**) Fourier analysis of the residuals in plot (**B**), with two overlapping dominating frequencies. (**F**) Fourier analysis of the residuals in plot (**C**). Positive oscillations represent vascular contraction, but, for COP, they can also imply the number of molecules with colloid strength that have been added to the plasma. Each circle in the top row (**A** to **C**) represents the measured plasma dilution and the solid line the optimal kinetic curve fit. Each circle in the bottom row (**D** to **F**) is the residual of the optimal kinetic curve fit and the solid line the oscillation wave according to the Fourier analysis.

Figure 2 and Figure 3 show examples of plasma dilution and plasma COP curves from which data on oscillatory patterns were retrieved.

### 3.2. Frequencies and Amplitudes

The dominating wave frequency was 151 ± 45, 141 ± 47, and 140 ± 36 min (mean ± SD) when the calculations were based on plasma dilution, plasma COP, and the plasma albumin concentration, respectively. The corresponding wave amplitudes were 1.57 ± 0.87%, 1.36 ± 0.56%, and 2.19 ± 0.93% (highest 10% percentiles being 3.0%, 2.0%, 3.4%, respectively). This dominating frequency constituted approximately 75% of the total residual variability in the curves, and 60% if the low frequencies were excluded. Two dominating frequencies in the same curve was very rare, but one such example is shown in Figure 3E.

Statistical analysis showed that the dominating wave frequency did not differ significantly depending on the measurement variable being plasma dilution, increase in COP, or plasma albumin (repeated-measures ANOVA). In contrast, the wave amplitudes were higher for albumin than for plasma dilution (*p* < 0.01), whereas COP scored slightly lower than plasma dilution (*p* < 0.01 vs. plasma albumin, Bonferroni correction used).

### 3.3. Subgroup Analysis

The data are presented according to their subgroups in Table 1. The wave amplitude based on plasma dilution differed depending on the subgroup (one-way ANOVA, *p* < 0.02); specifically, the amplitude was higher in the volunteers who received albumin 5% instead of albumin 20% (*p* < 0.024). The amplitudes differed when based on plasma albumin (*p* < 0.01) but, post hoc, we could not verify a difference between any specific subgroups. However, the two groups with inflammation, i.e., the post-burn and postoperative patients, collectively had higher wave amplitudes based on albumin than the non-inflammatory groups had collectively (*p* < 0.001). There were no statistically significant differences in amplitude between the subgroups when indicated by plasma COP (*p* = 0.093). A comparison between the inflammatory and non-inflammatory groups with regard to plasma COP was unsuccessful because this variable was not measured in the postoperative patients.

### 3.4. Correlations

**Figure 4:**
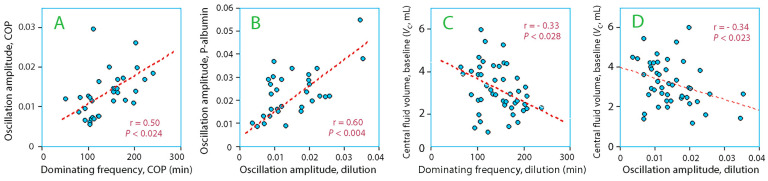
(**A**) Dominating oscillation frequency versus the corresponding amplitude, based on the measurements of the plasma colloid osmotic pressure (COP). (**B**) Oscillation amplitudes derived from the plasma dilution versus plasma albumin profiles. (**C**) Dominating oscillation frequency based on the measurements of plasma dilution versus the size of the central fluid volume (*V*_c_), derived via population volume kinetic analysis. (**D**) Oscillation amplitude based on the measurements of plasma dilution versus the size of the central fluid volume (*V*_c_). Each point represents one subject, and the hatched red lines are the regression lines. The infusion of albumin 5% are not included in subplots **C** and **D**.

The wave frequency correlated positively with the amplitude for plasma dilution (r = 0.31; *p* < 0.0257) and plasma COP (Figure 4A). Moreover, the amplitudes for plasma dilution and COP correlated with each other (Figure 4B).

The central body fluid volume (*V*_c_) in a volume kinetic analysis of the distribution of the infused fluid decreased with both the dominating frequency (Figure 4C) and the wave amplitude (Figure 4D).

### 3.5. Changes in Albumin Content

The product of plasma albumin and the Hb-derived plasma dilution was calculated to obtain the variations in intravascular albumin content, which suddenly increases as a sign of a lymphatic burst or an intensified lymphatic inflow. Such events (or periods) occurred at rates of 22% in the volunteers, 47% during surgery, 7% postoperatively, and 27% post-burn; all of these participants received albumin 20% (mean incidence, 21%). Sudden increases in the albumin content were also recorded in 17% of the volunteers receiving albumin 5%.

These events started after 154 ± 59 min and lasted 72 ± 40 min.

## 4. Discussion

### 4.1. Key Findings

Low-frequency oscillations occurred in the vascular system when albumin was given by intravenous infusion. They were often possible to discern with the naked eye. These oscillations represent changes in plasma volume that apparently occur at a consistent interval after a 30 min infusion of albumin. Fourier analysis showed that the frequency of the oscillatory waves was consistent at 2.3 h, regardless of study setting. Only a few recordings contained two dominating frequencies that overlapped. The amplitudes averaged 2% and their variability was also limited, although they were somewhat greater when based on plasma albumin.

### 4.2. Interpretation

A wave amplitude of 2% means that the plasma volume increases and decreases by 2% in a regular pattern. The “peak-to-peak” amplitude then amounts to 4%, which corresponds to 120 mL in an adult human, or to 25–30% of the maximally induced plasma volume expansion by the infused fluid. The highest percentiles of oscillations created even greater variability in the plasma volume (≈6%, 180 mL). Waves of this magnitude are likely to confound assessments of plasma volume and fluid responsiveness, which require the blood volume to be at a steady state and a predictable increase in cardiac preload in response to a bolus infusion of fluid.

### 4.3. Lymphatic Pumping

Post-infusion patterns mostly reflected changes in plasma volume, as the variations in plasma dilution and albumin followed the same pattern (Figure 4B). However, intravascular albumin mass increased suddenly or during some period in 21% of the participants, which is a sign of accelerated lymphatic flow. Efferent lymph contains 50% as much albumin as the plasma [15,16,17] but, in contrast to plasma water, its outflow from the vascular compartment by transcapillary leakage is highly controlled.

Long intervals between the oscillatory waves were associated with higher amplitudes (Figure 4A), which supports that a unifying pumping mechanism create them. Moreover, the lymphatic system seems to need a longer “rest” before the most forceful waves can be created. The lymphatic pumping activity is stimulated by norepinephrine [18,19], which was only given to the surgical patients; this group had the highest incidence of periods with increasing amounts of intravascular albumin. More than negligible amounts of this drug were only given to half of these patients, and the numbers were too low for statistical separation; however, the amplitude of the plasma dilution oscillations averaged 28% higher in those who received norepinephrine while the dominating wave frequency was only 4% longer, which indicates an inotrophic effect [20,21].

The wave amplitudes for plasma albumin were generally higher than for plasma dilution, which is consistent with the fact that lymph has a lower albumin concentration than plasma. The waves based on plasma albumin were highest in patients with inflammation, which might be due to the lymph containing even less albumin than usual. Alternatively, inflammation-associated endothelial injury [22,23] caused the capillary leakage of albumin to vary with the variations in hydrostatic pressure that the waves created.

The inverse relationship between the size of the central volume that expanded the infused fluid (*V*_c_) and both the oscillatory frequency (Figure 4C) and the wave amplitude (Figure 4D) further supports the theory the oscillations are created by an accelerated lymphatic flow. The reason is that *V*_c_ becomes small when fluid of unknown origin is added to the vascular system that is not a part of the modeled oncotic-driven recruitment of extravascular fluid. These correlations suggest that the longest dominating frequencies, where lymphatic pumping creates the highest wave amplitudes, persistently redistribute interstitial volume to the plasma. The equation *V*_c_ = X_d_/C_p_ relates to this situation; here, X_d_ is the dose and C_p_ the concentration, and *V*_c_ decreases if C_p_ increases without any change in X_d_ [24].

### 4.4. Literature

Oscillating rhythms are integral parts of human physiology [25] and oscillations in the vascular system have metabolic connections [26]. A previous study explored oscillations associated with crystalloid fluid therapy [2]. The wave frequency was multiples of 1 h. The amplitudes in volunteers were slightly lower than found here (1.3%) while high amplitudes (3.8%) were found in laparoscopic surgeries performed in the Trendelenburg position (20^o^ head down). A volume kinetic analysis was performed and showed that amplitudes > 1.5% were associated with an unusually fast flow of fluid through the interstitium, which also means that the flow of lymph to the plasma occurred at a higher rate than in subjects with a lower wave amplitude.

Other oscillatory patterns in the vascular system have been described. These include periodic pressure variations with the heartbeats (approximately 1 Hz) [27], the respiratory cycle (0.2 Hz), and with sympathetic (0.1 Hz) and parasympathetic activity (0.003 Hz) [1,28,29]. These cycles are likely to influence the movements of albumin in the body.

Oscillations also occur in the microcirculation. The identified types overlap those described above, but there is an additional oscillation that arises from the endothelium [30]. This has the lowest frequency of all, at 1–2 min; the waves created at the lower end of the range are dependent on nitric oxide (NO) [31], which is a molecule that plays a pivotal role in lymphatic pumping [32]. Therefore, NO might promote flow in both small blood and lymphatic vessels, although an impact on the plasma volume variation has not been clearly demonstrated previously.

### 4.5. Limitations

The limitations of this research include that only the most dominant oscillation was considered in the presentation, although there were occasionally two that overlapped. The long interval between the samplings (10 min) prevented high-frequency oscillations from being detected. The sampling period was usually 5 h [12,13,14] but extended to 6 h in 12 of the volunteers as well, as when albumin 5% was studied [11].

The number of analyses that did not fulfill the quality criteria for presentation was sometimes unevenly distributed between the variables (dilution, COP, and albumin) which hampered some statistical comparisons. Moreover, plasma COP was not measured when data were collected in association with surgery.

Oscillations were studied during fluid therapy. It is unclear whether the oscillations described here also occur without fluid volume loading. Moreover, no control method for the validation of the plasma volume changes and the subject’s dry weight was applied.

## 5. Conclusions

Oscillations derived from residual plots during infusion experiments with albumin solutions (mostly 20%) had a wave period of 2.3 h and an average amplitude of 2%. The top percentile showed a total amplitude (“peak-to-peak”) amounting to 6% of the plasma volume. The oscillations are probably due to variations in lymphatic pumping activity. They may confound measurements of plasma volume, fluid responsiveness, and cardiac output, which are best performed when the blood volume is at a steady state.

## Figures and Tables

**Table 1:** The wave frequency and amplitude of the oscillations recorded, depending on subgroup.

Study Group	Studied	Reported	Variable	Frequency (min)	Amplitude (%)	% of the Residual in Dominating Freq. with/Without Low Freq.	Reference nr
							
**Volunteers**	27	22	Dilution	150 ± 52	1.3 ± 0.6	75 ± 10/60 ± 8	11, 12
		17	COP	143 ± 45	1.3 ± 0.6	77 ± 12/60 ± 11	
		18	Albumin	132 ± 35	1.8 ± 0.6	78 ± 11/63 ± 15	
							
**Surgery**	15	8	Dilution	142 ± 39	1.5 ± 0.4	83 ± 5/63 ± 11	13
		8	Albumin	147 ± 24	2.0 ± 0.7	69 ± 6/62 ± 9	
							
**Postoperative**	15	5	Dilution	144 ± 39	1.2 ± 0.5	70 ± 8/57 ± 6	14
		7	Albumin	146 ± 41	2.8 ± 0.6	66 ± 7/58 ± 6	
							
**Post-burn**	15	10	Dilution	147 ± 45	1.9 ± 0.9	83 ± 9/63 ± 9	12
		12	COP	143 ± 40	1.2 ± 0.5	74 ± 12/60 ± 9	
		11	Albumin	151 ± 38	2.6 ± 1.5	75 ± 10/64 ± 11	
							
**5% albumin**	12	6	Dilution	128 ± 40	2.5 ± 1.5	79 ± 5/57 ± 6	11
		6	COP	126 ± 63	1.4 ± 0.8	90 ± 5/49 ± 16	
		7	Albumin	143 ± 43	1.6 ± 0.3	74 ± 7/58 ± 7	

Freq. = frequency; COP was not measured during surgery and the postoperative period.

## Data Availability

The original data sets and the Fourier analyses can be obtained from the author at reasonable request. The data used for the kinetic analysis are presented in Supplementary Material.xls. Further inquiries can be directed to the corresponding author at the following e-mail address: robert.hahn@ki.se.

## Supplementary Materials

The following supporting information can be downloaded at: https://www.mdpi.com/article/10.3390/life15050749/s1.
